# Isolating the Effect of Arch Architecture on Aortic Hemodynamics Late After Coarctation Repair: A Computational Study

**DOI:** 10.3389/fcvm.2022.855118

**Published:** 2022-06-24

**Authors:** Vahid Goodarzi Ardakani, Harshinee Goordoyal, Maria Victoria Ordonez, Froso Sophocleous, Stephanie Curtis, Radwa Bedair, Massimo Caputo, Alberto Gambaruto, Giovanni Biglino

**Affiliations:** ^1^Department of Mechanical Engineering, University of Bristol, Bristol, United Kingdom; ^2^University Hospitals Bristol and Weston, NHS Foundation Trust, Bristol, United Kingdom; ^3^Bristol Medical School, University of Bristol, Bristol, United Kingdom; ^4^National Heart and Lung Institute, Imperial College London, London, United Kingdom

**Keywords:** aortic coarctation, aortic hemodynamics, computational modeling, computational fluid dynamics, power loss, wall shear stress

## Abstract

**Objectives:**

Effective management of aortic coarctation (CoA) affects long-term cardiovascular outcomes. Full appreciation of CoA hemodynamics is important. This study aimed to analyze the relationship between aortic shape and hemodynamic parameters by means of computational simulations, purposely isolating the morphological variable.

**Methods:**

Computational simulations were run in three aortic models. MRI-derived aortic geometries were generated using a statistical shape modeling methodology. Starting from *n* = 108 patients, the mean aortic configuration was derived in patients without CoA (*n* = 37, “no-CoA”), with surgically repaired CoA (*n* = 58, “r-CoA”) and with unrepaired CoA (*n* = 13, “CoA”). As such, the aortic models represented average configurations for each scenario. Key hemodynamic parameters (i.e., pressure drop, aortic velocity, vorticity, wall shear stress WSS, and length and number of strong flow separations in the descending aorta) were measured in the three models at three time points (peak systole, end systole, end diastole).

**Results:**

Comparing no-CoA and CoA revealed substantial differences in all hemodynamic parameters. However, simulations revealed significant increases in vorticity at the site of CoA repair, higher WSS in the descending aorta and a 12% increase in power loss, in r-CoA compared to no-CoA, despite no clinically significant narrowing (CoA index >0.8) in the r-CoA model.

**Conclusions:**

Small alterations in aortic morphology impact on key hemodynamic indices. This may contribute to explaining phenomena such as persistent hypertension in the absence of any clinically significant narrowing. Whilst cardiovascular events in these patients may be related to hypertension, the role of arch geometry may be a contributory factor.

## Introduction

Aortic coarctation (CoA) is a discrete congenital heart defect with an incidence of 1/2,500 live births that can occur in isolation or in association with other left sided congenital lesions, such as bicuspid aortic valve (BAV) (1). The association between CoA and BAV is reported in up to 85% of CoA cases and has significant clinical implications on long-term outcomes, morbidity and mortality (1). Cardiovascular effects are mostly through new or persistent systemic hypertension; this may persist after surgery, which suggests that patients retain an abnormal vascular phenotype post-repair.

Aortic arch geometry patterns have been described as either “Gothic” (more angulated) or “Romanesque” (rounder) morphology. It has been suggested that arch geometry, together with increased arterial stiffness, contribute to the development of hypertension in CoA patients (2, 3). Abnormal aortic arch geometry has been associated with rest or exercise-induced hypertension in the long-term follow-up of patients after successful repair of CoA without residual obstruction of the arch (3–5). Hypertension has been linked to lower distensibility and elevated stiffness of the ascending aorta and greater loss of systolic wave amplitude across the aortic arch. This could partially explain the presence of late hypertension in the long-term follow-up of these patients, even in the absence of residual arch stenosis (6, 7). Furthermore, gothic arch geometry can unfavorably impact on left ventricular mass index (LVMI), independent of age and resting systolic blood pressure, suggesting a chronic increase in left ventricular afterload in the presence of gothic arch geometry (4).

The concept of “optimal surgical shape,” based on a three-dimensional (3D) analysis of aortic morphology in BAV patients with and without CoA, has been recently introduced into the literature. This showed that patients with repaired CoA were more likely to have a gothic aortic arch morphology and this has been previously associated with new or persistent systemic hypertension in the long term (8). Furthermore, the “selfish brain” hypothesis was recently described, showing that vertebral artery hypoplasia with an incomplete posterior circle of Willis in repaired CoA subjects may be important in the development of hypertension or its persistence, following CoA repair (9). This is suggestive of a more global vascular developmental phenomenon, rather than simple hemodynamics.

Undoubtedly, CoA leads to long-term ventricular and arterial implications and cardiovascular outcomes, with these patients presenting significantly lower survival than the normal population (10), even in the modern era. Therefore, a thorough understanding of CoA hemodynamics and their ramifications is important. The motivation of the study is indeed to make use of computational modeling and the insight they offer in terms of isolating a variable of interest (in this case, aortic arch shape) to contribute to the understanding of aortic pressure changes in CoA patients. The broad clinical relevance of this approach is not only related to the observation that hypertension may be an inevitable consequence of CoA, even following early effective anatomical repair, but that maladaptive processes may be at play in these patients (11). Also in the context of interventional rather than surgical repair, the importance of the aortic arch anatomy in CoA patients is increasingly recognized as a parameter to define cases at higher risk of residual hypertension, even despite optimized isthmic stent implantation (12).

This work focuses purely on fluidodynamic aspects, aiming to analyze the relationship between aortic shape and hemodynamic parameters by means of computational fluid dynamics, purposely isolating the morphological variable and comparing patients with successfully repaired CoA, with unrepaired CoA and without CoA.

## Methods

### Aortic Arch Models

**Figure 1:**
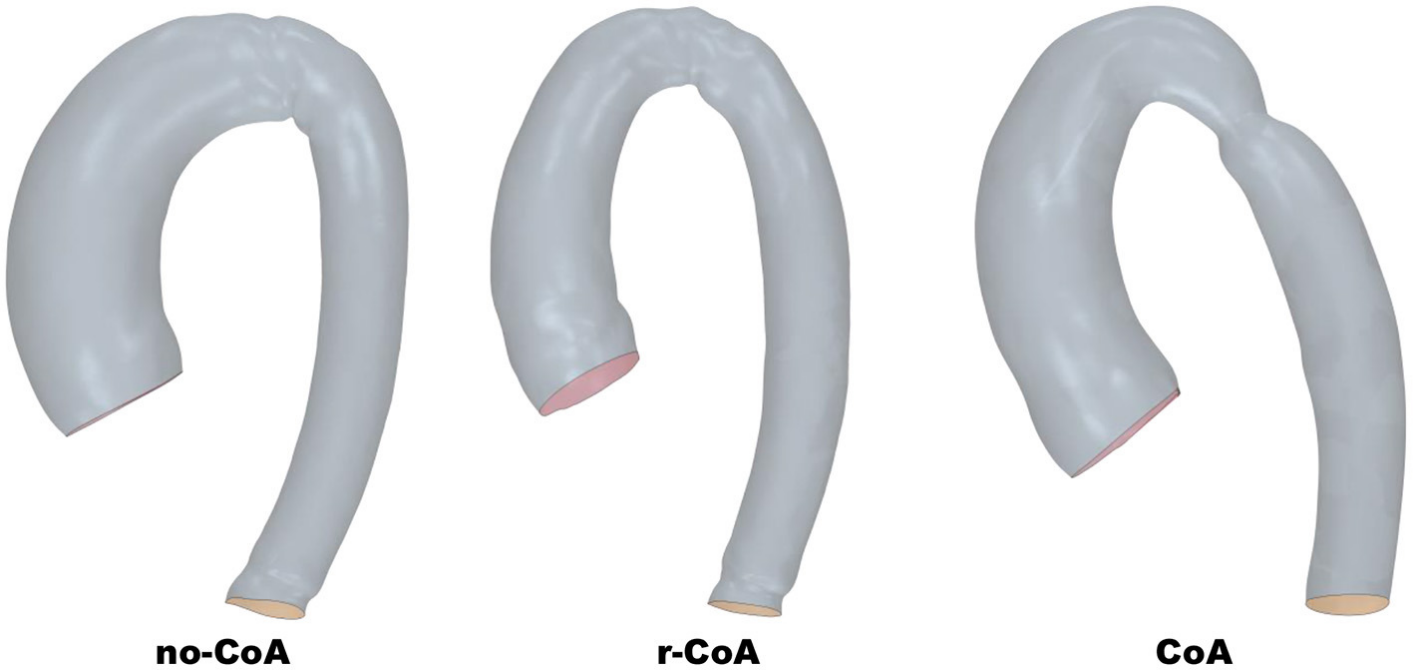
Aortic geometries derived from statistical shape modeling.

The study is based on three aortic models derived from a previously described population of patients with BAV and CoA (8). Starting from *n* = 108 patients, the mean aortic arch configuration was derived in patients without CoA (*n* = 37, “No-CoA”), with surgically repaired CoA (*n* = 58, “r-CoA”) and with unrepaired CoA (*n* = 13, “CoA”). The aortic shapes were reconstructed from magnetic resonance imaging (MRI) data and the final three configurations were obtained by means of statistical shape modeling, thus producing three average models summarizing the geometrical features of the patients in each group. This resulted in three representative models for the three scenarios of interest (Figure 1). More details on the process of image reconstruction are available in (8) and details on the statistical shape modeling framework are available in (13). All datasets were anonymized and, in view of the retrospective study design, formal ethical approval was waived by the local Institutional Research and Innovation Department, and the rest of the study focused on computational simulations.

### Computational Fluid Dynamics

Computational simulations were run in the three aortic models, purposely isolating the geo-metrical variable. The mesh generation and the numerical simulation were both carried out in STAR-CCM+ 13.04.010-r8 (Siemens). The flow was simulated using an unsteady implicit scheme, with the SIMPLE algorithm to solve the pressure and velocity in a segregated manner. Second order accuracy was chosen for both temporal and spatial discretization. The time step size and the convergence criteria were set to be *t* = 0.001 s and ε = 10^−8^, respectively. The fluid was assumed to be incompressible and Newtonian, and the density and dynamic viscosity were set to ρ = 1, 060 kg/m^3^ and μ = 0.004 Pa.s, respectively.

**Figure 2:**
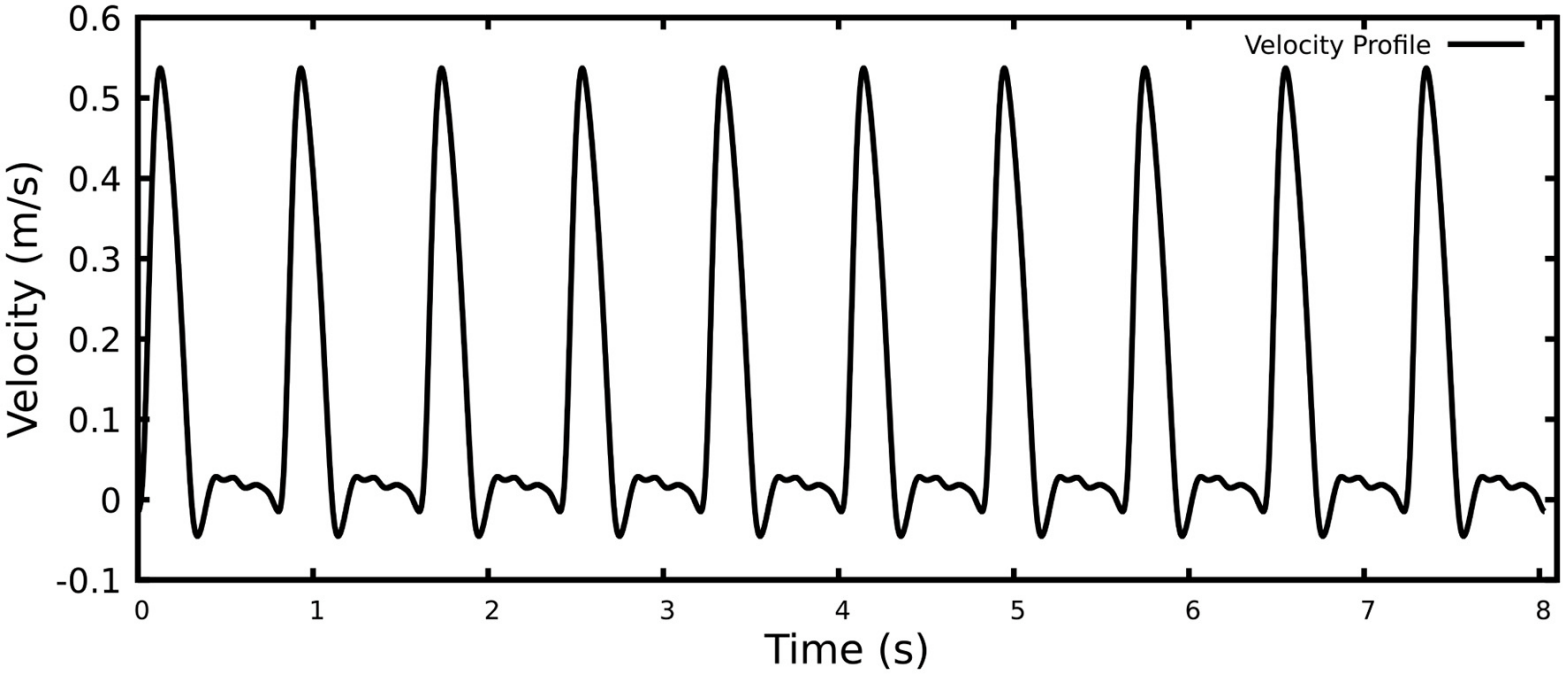
Realistic pulsatile velocity profile for 10 heartbeats.

A polyhedral computational grid together with 10 prism layers was generated with an average total number of 6 M elements for the finite volume solver. In order to capture complex flow features as much as possible, the flow was modeled using Large Eddy Simulation (LES), more specifically Dynamic Smagorinsky Subgrid Scale. Literature has suggested that this model is reasonably capable of simulating aortic flows (14–16). A realistic aortic flow waveform derived from MRI (Figure 2) was prescribed as the inlet velocity profile, with average flow rate of 6 L/min, peak velocity of 53.7 cm/s and average velocity of 11.7 cm/s. The outlet boundary condition was considered to be zero pressure. Ten cardiac cycles were simulated and only the last one was used for subsequent analyzes.

**Figure 3:**
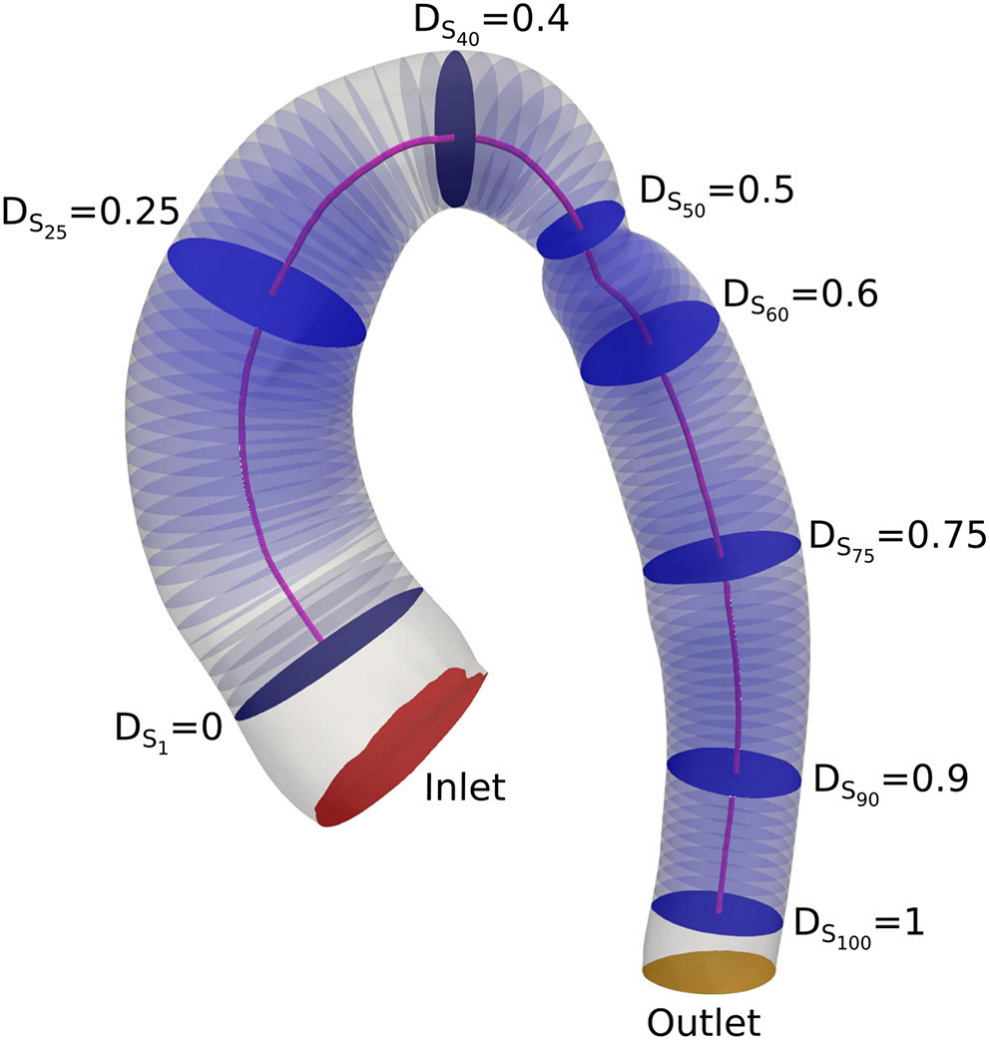
Location of the cross-sections and the centerline.

In order to compare the computed hemodynamic results with respect to geometric characteristics of the aortic arch, the medial axis of each geometry was computed using Vascular Modeling Toolkit (VMTK) (17). One-hundred cross-sections along the aorta were further generated such that their normal vectors were locally tangent to the centerline. The average of flow parameters of interest (i.e., pressure, velocity, wall shear stress, vorticity) were measured on each cross-section along the aorta. The slices at positions 40, 50, and 60 were located before, across, and after the coarctation, respectively, and were selected for direct comparison between the different geometries. The slice at position 90 was also included to capture the effect of coarctation on the blood flow properties downstream of the narrowing, in the descending aorta. The location of the slices was normalized from 0 to 1, and is displayed in Figure 3.

Comparisons across the three aortic configurations were carried out at three time points, i.e., peak systole (0.13 s, 16% of the cardiac cycle), end systole (0.34 s, 43% of the cardiac cycle) and end diastole (0.80 s, 100% of the cardiac cycle). Key measurements included velocity (m/s, derived directly from the simulations), pressure drop (mmHg, measured as *P(S*_0.9_*)–P(S*_0.4_*)*, accounting for pressure recovery) and vorticity (defined as the curl of the velocity, which is a measure of the rotation of the flow, /s). Wall shear stress (WSS, *dyn*/*cm*^2^) was also derived from the simulations. In order to identify areas of elevated WSS and draw comparisons between the three geometries, the average of the obtained WSS was calculated and areas with values higher than this were considered as elevated WSS. The length and number of strong separations in the descending aorta were also investigated by comparing velocity streamlines along the aortic arches.

### Data Analysis

Results were compared for the three aortic configurations at the above mentioned three time instances along the length of the aorta. Whilst the aortic models summarize the geometrical features of dozens of patients, the comparison was ultimately carried out on the three statistically derived shapes. This precluded a full statistical analysis, which was beyond the scope of this study, rather taking advantage of the fact that the representative aortic geometries in themselves summarize features from multiple patients.

## Results

Velocity, pressure and vorticity values for all geometries and time points are reported in Table 1.

**Table 1:** Average values of velocity (V), pressure (P), and vorticity (ω) on slices of 40, 50, 60, and 90.

		**No-CoA**			**r-CoA**			**CoA**		
		**PS**	**ES**	**ED**	**PS**	**ES**	**ED**	**PS**	**ES**	**ED**
*S*_40_	V (m/s)	1.12 ± 0.3	0.13 ± 0.09	0.04 ± 0.01	1.57 ± 0.33	0.16 ± 0.07	0.05 ± 0.02	1.58 ± 0.05	0.17 ± 0.09	0.06 ± 0.03
	P (mmHg)	7.94 ± 1.94	−0.66 ± 0.1	−0.62 ± 0.002	8.08 ± 2.57	−1 ± 0.12	−0.66 ± 0.005	38.02 ± 7.19	−0.52 ± 0.1	−0.75 ± 0.007
	ω (/s)	252.5 ± 68	101.1 ± 120	17.11 ± 22	460.1 ± 1,211	129.8 ± 120	27.5 ± 31	458.8 ± 1,473	99.5 ± 137	32.9 ± 39
*S*_50_	V (m/s)	1.14 ± 0.44	0.28 ± 0.13	0.04 ± 0.02	1.47 ± 0.62	0.29 ± 0.11	0.05 ± 0.02	3.61 ± 0.65	0.36 ± 0.21	0.11 ± 0.04
	P (mmHg)	4.55 ± 2.91	−0.97 ± 0.16	−0.52 ± 0.003	0.6 ± 3.51	−0.96 ± 0.26	−0.5 ± 0.006	−9.38 ± 4.52	−0.73 ± 0.23	−0.6 ± 0.03
	ω (/s)	386.9 ± 647	204.2 ± 157	24.04 ± 20	710.6 ± 991	289.2 ± 190	27.76 ± 24	1,495.14 ± 4,573	284 ± 376	75 ± 98
*S*_60_	V (m/s)	1.13 ± 0.26	0.23 ± 0.13	0.06 ± 0.03	0.97 ± 0.31	0.32 ± 0.13	0.07 ± 0.03	1.78 ± 1.43	0.29 ± 0.13	0.07 ± 0.03
	P (mmHg)	5.51 ± 0.83	−0.74 ± 0.19	−0.44 ± 0.01	7.93 ± 0.65	−1.1 ± 0.28	−0.41 ± 0.01	−16.09 ± 2.44	−0.47 ± 0.24	−0.44 ± 0.01
	ω (/s)	250.7 ± 502	172.7 ± 173	39.63 ± 27	220.3 ± 320	285.3 ± 227	41.58 ± 26	1,077.7 ± 1,507	326.4 ± 232	45.94 ± 33
*S*_90_	V (m/s)	1.33 ± 0.28	0.19 ± 0.09	0.05 ± 0.02	1.14 ± 0.25	0.2 ± 0.09	0.06 ± 0.02	1.43 ± 0.3	0.27 ± 0.1	0.05 ± 0.02
	P (mmHg)	2.74 ± 0.6	−0.29 ± 0.08	−0.16 ± 0.005	6.01 ± 0.37	−0.36 ± 0.09	−0.15 ± 0.007	1.52 ± 0.51	−0.38 ± 0.24	−0.17 ± 0.007
	ω (/s)	272.8 ± 552	158.5 ± 100	30.87 ± 24	238.4 ± 469	210.5 ± 136	27.24 ± 21	320.7 ± 686	320.9 ± 237	34.27 ± 24

*PS, ES, ED stand for Peak Systole, End Systole, and End Diastole, respectively. Standard deviation is reported as the confidence interval. The location of slices is illustrated in Figure 3*.

**Figure 4:**
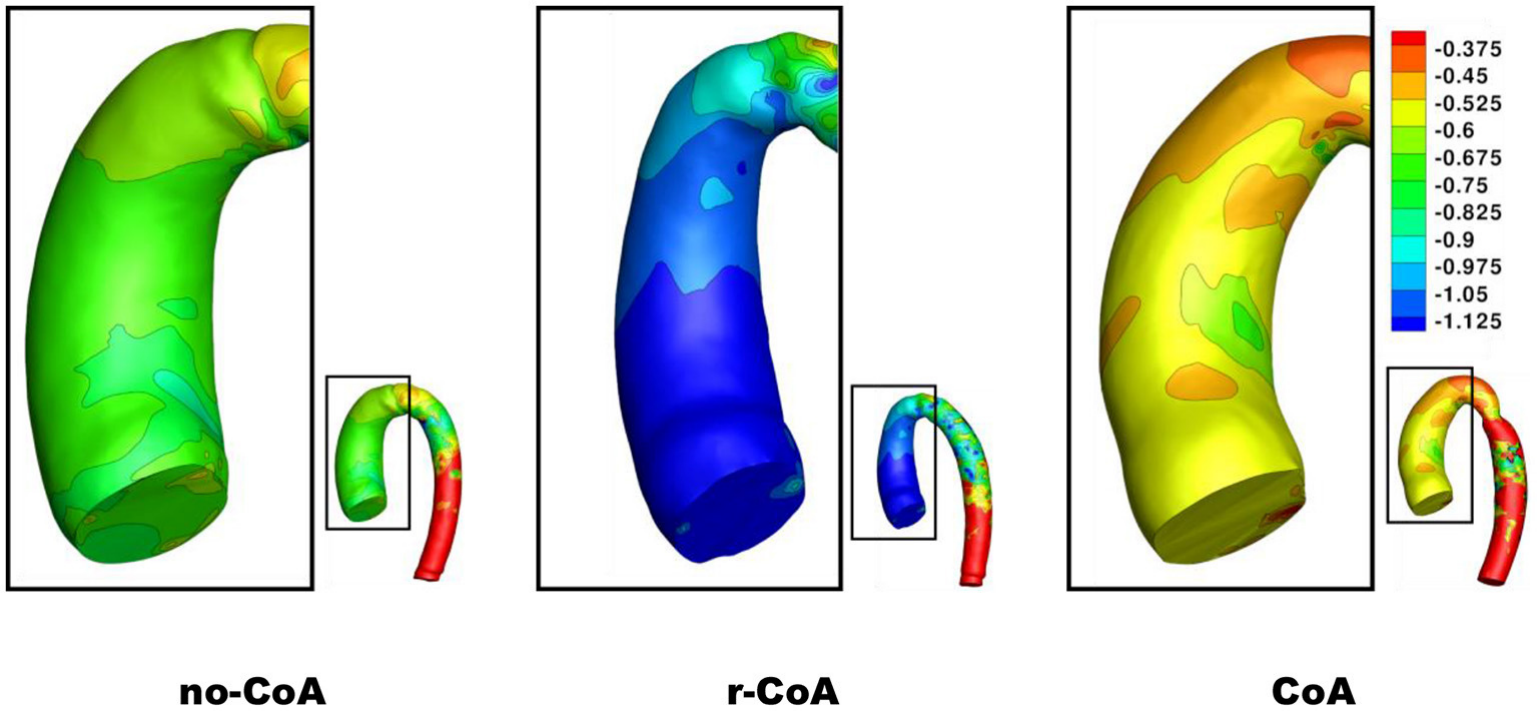
Contour of pressure (mmHg) on the ascending aorta at end systole.

**Figure 5:**
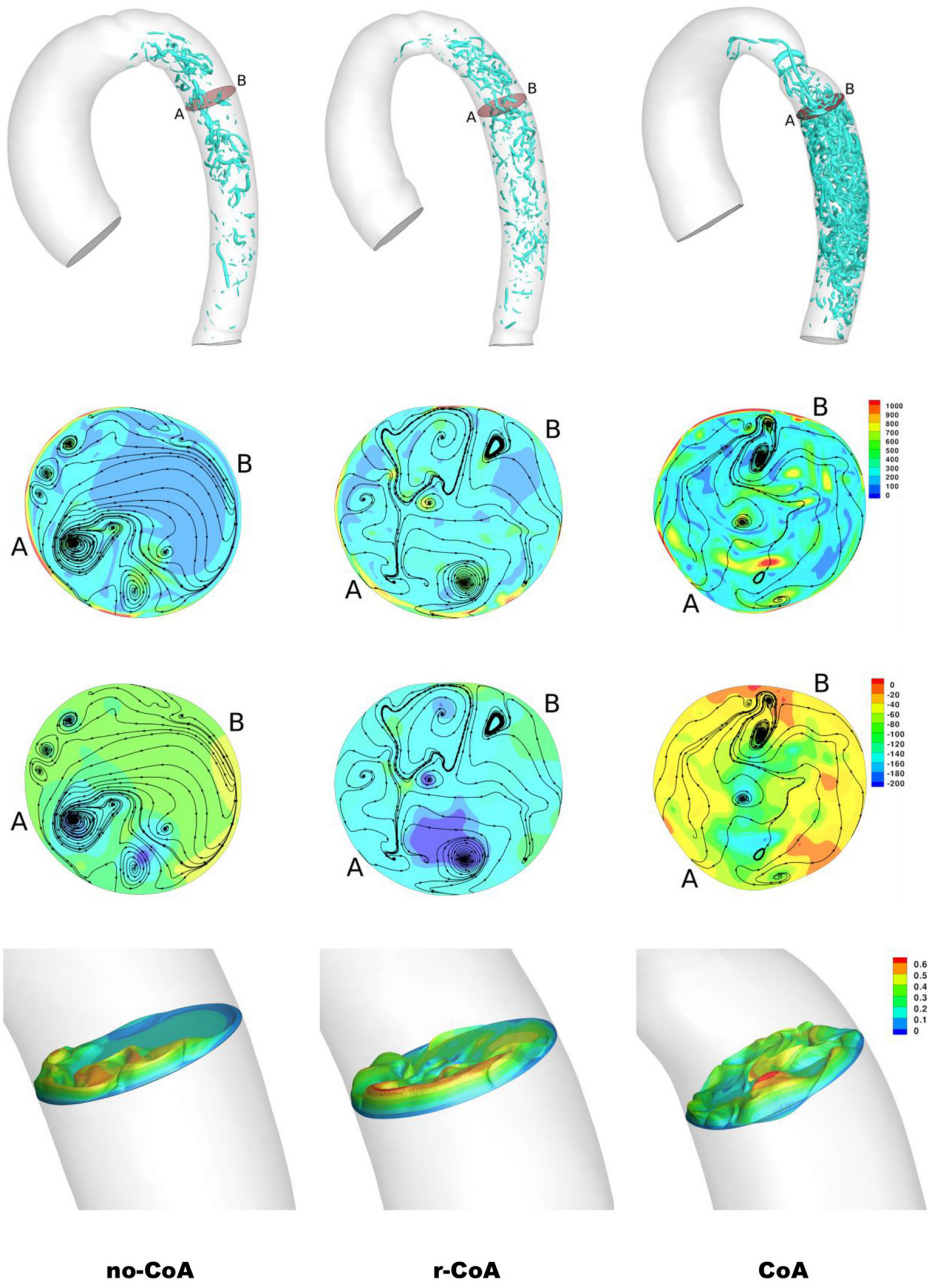
Result of the simulations at end systole. First row: vortical structure by iso-surface of λ_2_ = −30, 000 in the descending aorta. Second row: contour of vorticity (/s) along with velocity streamlines. Third row: contour of pressure along with velocity streamlines. Fourth row: velocity cross-section (m/s). The cross-sections are identified in the first row.

**Figure 6:**
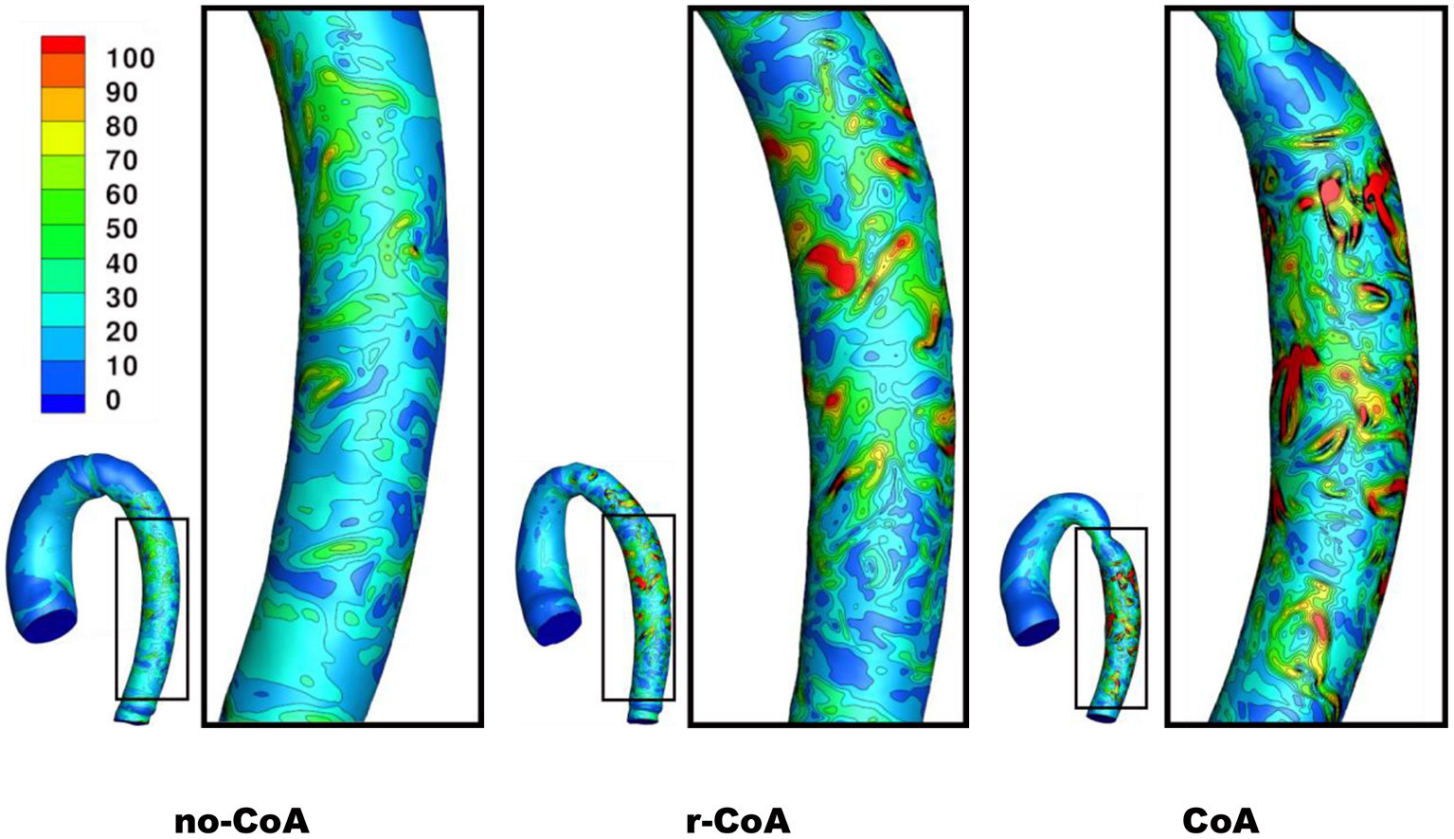
Contour of wall shear stress (WSS) on the descending aorta at end systole.

**Figure 7:**
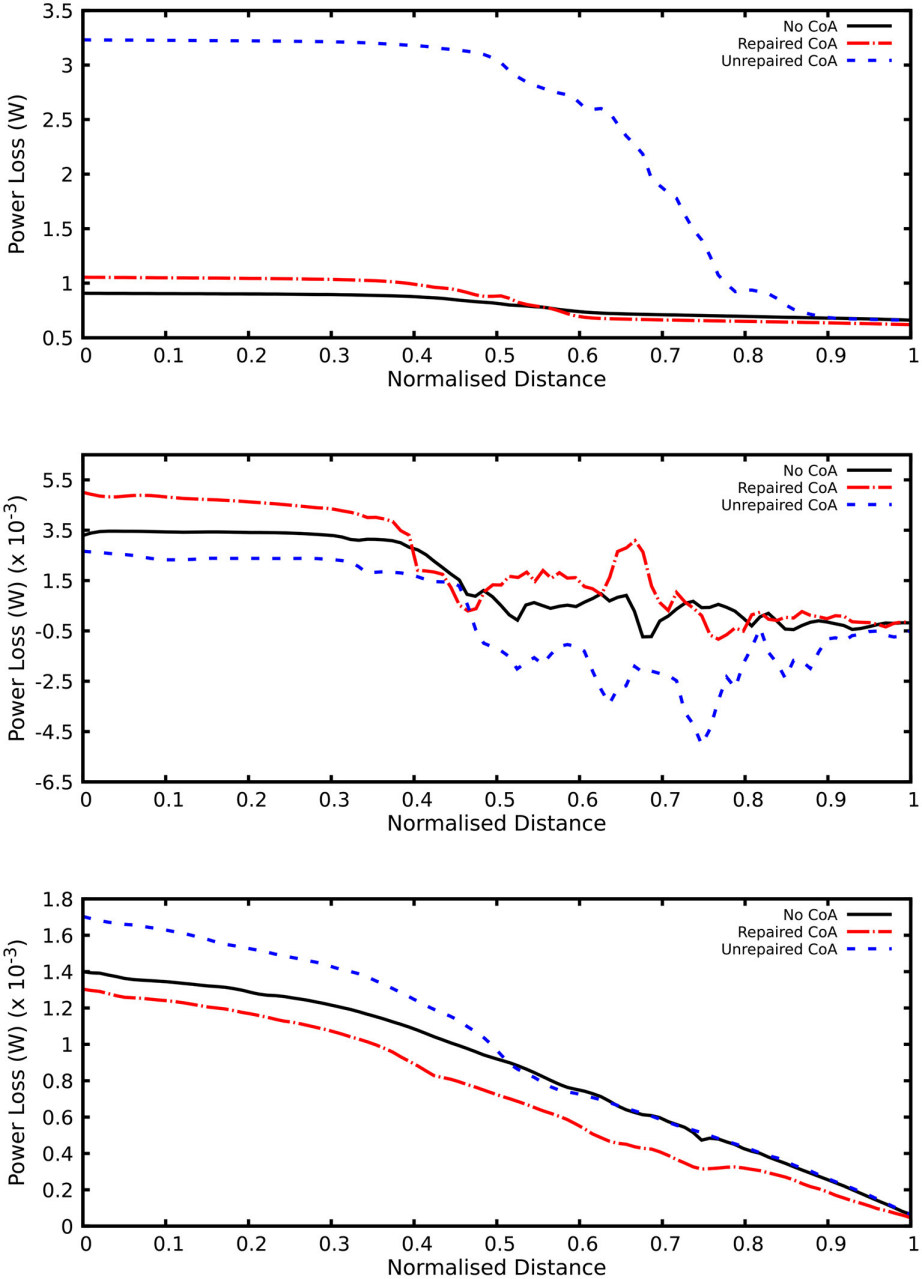
Plot of power loss along the aorta. Order of plots from top to bottom: end systole, peak systole, and end diastole.

Unsurprisingly, comparison between the no-CoA and the CoA models revealed much higher aortic pressure in the ascending aorta (four-fold increase at peak systole) and a substantial pressure drop across the coarctation site (5.20 vs. 36.50 mmHg; Figure 4) in the CoA group, as well as much higher aortic velocity past the CoA (1.14 vs. 3.61 m/s). Vorticity showed approximately a four-fold increase between the two configurations in the descending aorta (387 vs. 1,495 1/s at location 0.5 and 251 vs. 1,078 1/s at location 0.6). Vortex cores and vorticity across the aortic cross-section in the descending aorta are shown in Figure 5. Given the obtained WSS maps (Figure 6), areas of elevated WSS were identified (WSS >50 *dyn*/*cm*^2^) and noted in the descending aorta and no-CoA and CoA configurations were compared. There was an average 378% increase in the CoA group. Finally, a three-fold increase in power loss was observed at peak systole in the ascending aorta in the CoA group compared to the no-CoA group (0.9 vs. 3.2 W, No-CoA vs. CoA; Figure 7).

More interestingly, differences were also observed when comparing the no-CoA and r-CoA configurations, despite the latter representing qualitatively a successful CoA repair with a coarctation index >0.8 (i.e., no significant narrowing from a clinical standpoint). The hemodynamic observations in the r-CoA were mainly:

1) increased vorticity at the site of CoA repair (no-CoA: 387 1/s vs. r-CoA: 711 1/s at location 0.5), Figure 5;
2) regions of elevated WSS in the descending aorta of the r-CoA model more comparable to the CoA configuration (Figure 6), with an increase in areas of WSS (>50 *dyn*/*cm*^2^) in 153% of the r-CoA model;
3) increased power loss (+22%) in the ascending aorta (no-CoA: 0.9 W vs. r-CoA: 1.1 W; Figure 7).

**Figure 8:**
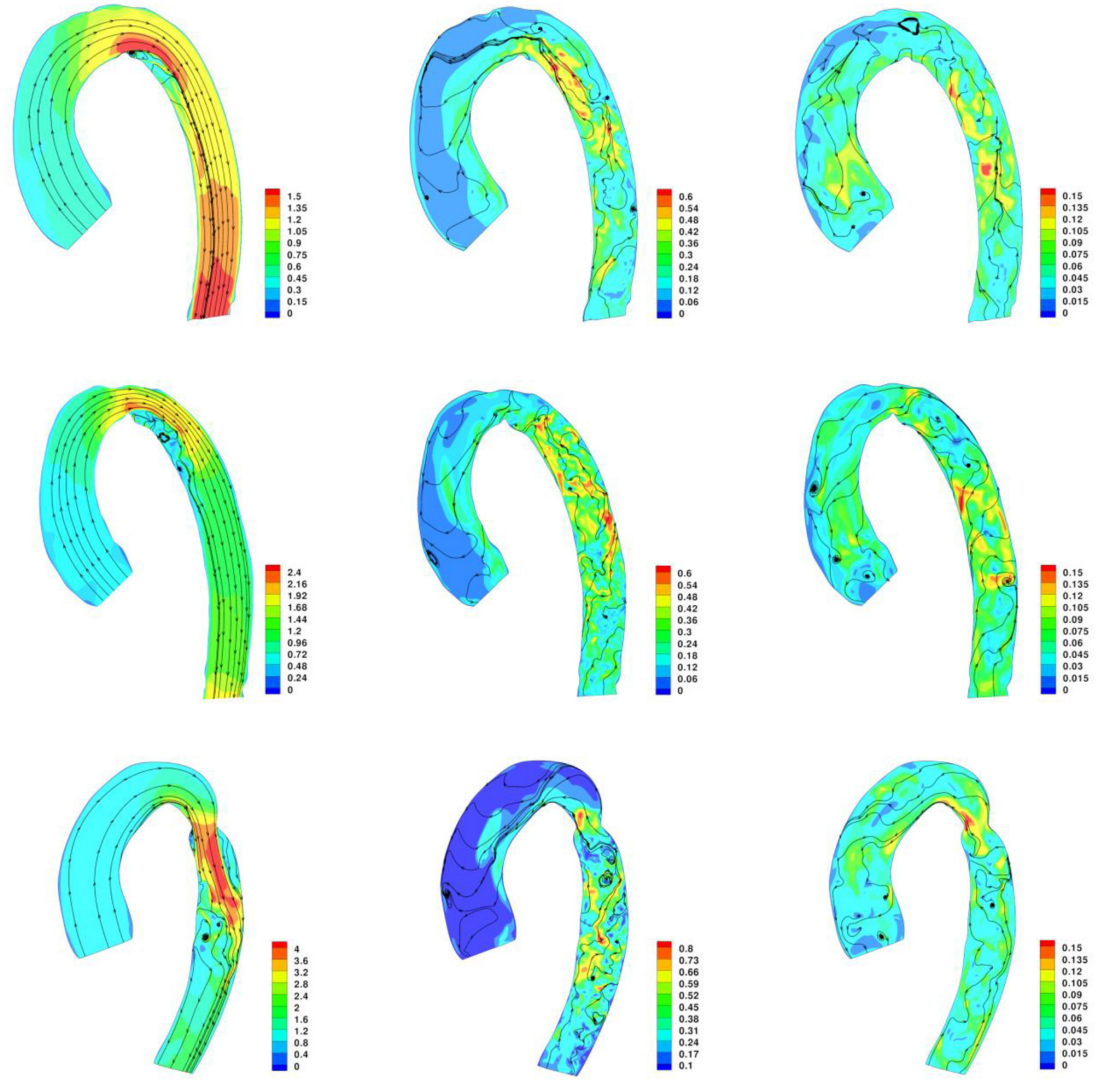
Contour of velocity and velocity streamlines along the aorta at peak systole, end systole, and end diastole. Top row: No-CoA aorta; Middle row: r-CoA; Bottom Row: CoA.

The velocity streamlines along the three aortic models are plotted in Figure 8. Two internal and external separations in the CoA aorta can be appreciated, leading to adverse pressure gradient in the descending aorta to such an extent that the average pressure in cross-sections *S*_0.5_ and *S*_0.6_ becomes negative at peak systole, as reported in Table 1. Internal separation was only observed in no-CoA and r-CoA aorta. The length of separations was measured and is reported in Table 2.

**Table 2:** Approximate length of separations in the three aortic configurations at peak systole.

	**Separation length (cm)**	
	**Internal**	**External**
No-CoA	1	–
r-CoA	1.5	–
CoA	1	2.6

## Discussion

Computational simulations have been providing an accessible and versatile tool for gathering insight into the fluid dynamics of aortic coarctation for over a decade. Computational modeling analysis suggests that the concomitant presence of BAV with CoA results in increased maximal velocity, secondary flow, pressure loss, time-averaged wall shear stress and oscillatory shear index in the descending aorta, past the CoA, compared to a tricuspid valve scenario (18). Computational fluid dynamics can highlight regions of the thoracic aorta with unfavorable hemodynamics (19) and, in the presence of CoA, reveal differences in local WSS (20). Such insight can yield clinically relevant messages. For instance, WSS changes can be related to areas of plaque formation in locations influenced by surgical CoA repair, such as resection with end-to-end anastomosis (19). The modeling literature suggests that simulations can be used to assess changes in aortic wall biomechanics after a percutaneous procedure (e.g., stenting) (21), to detect unfavorable flow patterns in patients with re-CoA (22), and to plan interventions in complex scenarios (23). In the present study, a computational approach was chosen to isolate the effect of aortic arch geometry (based on representative anatomical configurations of the aortic arch derived from a statistical shape modeling framework, rather than individual patients' anatomies) and offer new insights into the hemodynamic effect of aortic arch architecture, particularly in the presence of a repaired CoA. Simulations revealed interesting differences in the repaired CoA setting. The idealized and purposely simplified scenario adopted here in the CFD simulations (i.e., same inflow, no aortic wall deformation) allowed us to isolate the effect of the geometry of CoA repair and determine the impact of that variable. Hemodynamic differences mainly included a nearly doubled value of vorticity in the descending aorta past the repaired CoA region, increased WSS in the descending aorta, a mild increase in pressure drop compared to the isolated BAV scenario, and an increase in power loss.

These findings suggest that even in a successfully repaired CoA (i.e., no residual narrowing with a clinically negligible CoA index and a qualitatively optimal aortic arch geometry), there are important changes in the architecture of the arch. The elevated WSS in the descending aorta may relate to some degree of dilation in this area that has been previously observed in a study focusing on aortic arch morphology in BAV with/without CoA (8). In addition, power loss (which is a function of the pressure drop and the aortic flow rate) was also found to be different in BAV patients with r-CoA compared to patients with isolated BAV. Power loss was higher in the presence of CoA (both repaired and unrepaired). This concurs with existing literature reporting an association between increasing stenosis level and increasing energy dissipation (24). Furthermore, previous work in other CHD, such as the geometry of the total cavopulmonary connection in Fontan patients, has shown that power loss increases dramatically in a non-linear fashion with increasing cardiac output and is dependent on geometrical variables, suggesting the importance of studying exercise conditions as well as rest conditions (25). While our simplified model focused on isolating differences related to aortic arch morphology, the observed differences in power loss, especially in the r-CoA scenario, warrant further study and extension to the investigation of the effect of exercise physiology, as this is likely to exacerbate any differences. Another scenario that would be very relevant to include in such an analysis would be the case of transverse arch hypoplasia, particularly in light of recent work, based on principal component analysis (PCA), suggesting that arch morphology is not the major determinant of vascular load following coarctation repair and that caliber is more important than curvature (7). These observations are purely geometric and did not consider effects on the flow field, so they can be viewed as complementary alongside those presented in this article, contributing to refining our knowledge of CoA. Additionally, if a growth model of the aorta with CoA was developed following a recent methodology (26), it would be interesting to include factors such as recurrent arch obstruction, which is more prevalent in patients with a CoA index <0.7, extending the observations from this study.

The flow separation results suggest that small variations in the morphology of the aortic arch (native or resulting from surgical repair) lead to changes in flow velocity acceleration and also to changes in WSS in the descending aorta. Our observations agree with previous literature suggesting a relation between geometric irregularities and flow separation at the corners and that this increases from romanesque to crenel to gothic arches (27). It was particularly interesting to observe flow separation in the scenario of successful CoA repair with no residual significant narrowing, which reinforces previous observations that even when recreating a favorable anatomy after CoA repair, aortic hemodynamics are still not fully restored (28).

This study indeed reinforces observations that CoA repair does not restore normal hemodynamics, even if there is no residual narrowing. These patients could be assumed to have an abnormal vasculature from birth, regardless of repair with on-going cardiovascular risk, even in the absence of residual narrowing or systemic hypertension. It is crucially important to pay attention to the control of cardiovascular risk factors throughout life, such as refraining from smoking, actively managing elevated blood pressure and cholesterol and the encouragement of a healthy weight and exercise habits from an early age. Certain arch phenotypes may be most at risk and warrant intensive preventative therapy.

There are limitations to this study. The aortic models were set as rigid and the same realistic aortic inflow was used as a boundary condition for all three geometrical configurations. Whilst this was purposely done in order to isolate the geometrical variable, aortic distensibility and patient-specific flow features contribute to the hemodynamic changes in these patients. We suggest that we can further augment our knowledge of CoA hemodynamics by studying these variables in a parametric fashion in future studies. Furthermore, differences in relevant parameters such as WSS are likely to be affected by changes in the patients' physiological state, i.e., rest vs. exercise (28), which was not modeled in this study. Previous MRI-based analysis suggesting that transverse arch and isthmus hypoplasia rather than acute aortic arch angulation are involved in the pathophysiology of blood pressure response to peak exercise after CoA repair (29) and it has been observed that CoA patients without significant obstruction had higher exercise-induced systolic blood pressure changes than matched controls (30). Finally, whilst this study focused on aortic hemodynamics, it is important to note that CFD simulations in this context can also allow the comparison of relevant indices between the aorta and the brachiocephalic vessels, which is an important consideration in CoA patients, given the incidence of cerebrovascular events (28, 31). Whilst events may be related to hypertension, the role of arch geometry and anatomy of the head and neck vessels may well be a contributory factor.

## Conclusion

This study adds to our appreciation of aortic hemodynamics in patients with repaired aortic coarctation, suggesting that even small alterations in the aortic morphology (such as those resulting following surgical repair) impact on key hemodynamic indices. This can in part contribute to explaining phenomena such as persistent hypertension even in the absence of any clinically significant narrowing.

## Data Availability Statement

The raw data supporting the conclusions of this article will be made available by the authors, without undue reservation.

## Ethics Statement

Ethical review and approval was not required for the study on human participants in accordance with the local legislation and institutional requirements. The patients/participants provided their written informed consent to participate in this study.

## Author Contributions

VGA, MO, AG, and GB conceived the study. VGA and HG ran the computational simulations. FS provided the anatomical models. VGA and GB drafted the manuscript. All authors contributed to data interpretation and critically revising.

## Conflict of Interest

The authors declare that the research was conducted in the absence of any commercial or financial relationships that could be construed as a potential conflict of interest.

## Publisher's Note

All claims expressed in this article are solely those of the authors and do not necessarily represent those of their affiliated organizations, or those of the publisher, the editors and the reviewers. Any product that may be evaluated in this article, or claim that may be made by its manufacturer, is not guaranteed or endorsed by the publisher.

## References

[1] Kaemmerer H. Aortic coarctation and interrupted aortic arch. In: Gatzoulis MA, Webb GD, Daubeney PEF, editors. Diagnosis and Management of Adult Congenital Heart Disease. 2nd ed. Churchill Livingstone (2011). p. 261–70. 10.1016/B978-0-7020-3426-8.00036-8

[2] Ou P Celermajer DS Raisky O Jolivet O Buyens F Herment A. Angular (gothic) aortic arch leads to enhanced systolic wave reflection, central aortic stiffness, and increased left ventricular mass late after aortic coarctation repair: evaluation with magnetic resonance flow mapping. J Thorac Cardiovasc Surg. (2008) 135:62–8. 10.1016/j.jtcvs.2007.03.059 18179920

[3] Ou P Celermajer DS Mousseaux E Giron A Aggoun Y Szezepanski I. Vascular remodeling after “successful” repair of coarctation: impact of aortic arch geometry. J Am Coll Cardiol. (2007) 49:883–90. 10.1016/j.jacc.2006.10.057 17320747

[4] Ou P Bonnet D Auriacombe L Pedroni E Balleux F Sidi D. Late systemic hypertension and aortic arch geometry after successful repair of coarctation of the aorta. Eur Heart J. (2004) 25:1853–59. 10.1016/j.ehj.2004.07.021 15474701

[5] de Divitiis M Rubba P Calabrò R. Arterial hypertension and cardiovascular prognosis after successful repair of aortic coarctation: a clinical model for the study of vascular function. Nutr Metab Cardiovasc Dis. (2005) 15:382–94. 10.1016/j.numecd.2005.08.002 16216725

[6] Donazzan L Crepaz R Stuefer J Stellin G. Abnormalities of aortic arch shape, central aortic flow dynamics, and distensibility pre-dispose to hypertension after successful repair of aortic coarctation. World J Pediatr Congenit Heart Surg. (2014) 5:546–53. 10.1177/2150135114551028 25324252

[7] Quail MA Segers P Steeden JA Muthurangu V. The aorta after coarctation repair–effects of calibre and curvature on arterial haemodynamics. J Cardiovasc Magn Res. (2019) 21:1–9. 10.1186/s12968-019-0534-7 30975162 PMC6458643

[8] Sophocleous F Biffi B Milano EG Bruse J Caputo M Rajakaruna C. Aortic morphological variability in patients with bicuspid aortic valve and aortic coarctation. Eur J Cardiothorac Surg. (2019) 55:704–13. 10.1093/ejcts/ezy339 30380029 PMC6459283

[9] Rodrigues JC Jaring MF Werndle MC Mitrousi K Lyen SM Nightingale AK. Repaired coarctation of the aorta, persistent arterial hypertension and the selfish brain. J Cardiovasc Magn Reson. (2019) 21:1–10. 10.1186/s12968-019-0578-8 31703697 PMC6839237

[10] Lee MG Babu-Narayan SV Kempny A Uebing A Montanaro C Shore DF. Long-term mortality and cardiovascular burden for adult survivors of coarctation of the aorta. Heart. (2019) 105:1190–96. 10.1136/heartjnl-2018-314257 30923175

[11] Kenny D Polson JW Martin RP Paton JFR Wolf AR. Hypertension and coarctation of the aorta: an inevitable consequence of developmental pathophysiology. Hypertens Res. (2011) 34:543–7. 10.1038/hr.2011.22 21412243

[12] Iriart X Laik J Cremer A Martin C Pillois X Jalal Z. Predictive factors for residual hypertension following aortic coarctation stenting. J Clin Hypertens. (2019) 21:291–8. 10.1111/jch.13452 30585428 PMC8030514

[13] Bruse JL McLeod K Biglino G Ntsinjana HN Capelli C Hsia T-Y. A statistical shape modelling framework to extract 3d shape biomarkers from medical imaging data: assessing arch morphology of repaired coarctation of the aorta. BMC Med Imaging. (2016) 16:1–19. 10.1186/s12880-016-0142-z 27245048 PMC4894556

[14] Tan F Wood N Tabor G Xu X. Comparison of LES of steady transitional flow in an idealized stenosed axisymmetric artery model with a RANS transitional model. J Biomech Eng. (2011) 133:051001. 10.1115/1.4003782 21599092

[15] Varghese SS Frankel SH Fischer PF. Modeling transition to turbulence in eccentric stenotic flows. J Biomech Eng. (2008) 130:014503. 10.1115/1.2800832 18298194

[16] Mittal R Simmons S Udaykumar H. Application of large-eddy simulation to the study of pulsatile flow in a modeled arterial stenosis. J Biomech Eng. (2001) 123:325–32. 10.1115/1.1385840 11563757

[17] Antiga L Piccinelli M Botti L Ene-Iordache B Remuzzi A Steinman DA. An image-based modeling framework for patient-specific computational hemodynamics. Med Biol Eng Comput. (2008) 46:1097–112. 10.1007/s11517-008-0420-1 19002516

[18] Keshavarz-Motamed Z Garcia J Kadem L. Fluid dynamics of coarctation of the aorta and effect of bicuspid aortic valve. PLoS ONE. (2013) 8:e72394. 10.1371/journal.pone.0072394 24015239 PMC3754982

[19] Wendell DC Samyn MM Cava JR Ellwein LM Krolikowski MM Gandy KL. Including aortic valve morphology in computational fluid dynamics simulations: initial findings and application to aortic coarctation. Med Eng Phys. (2013) 35:723–35. 10.1016/j.medengphy.2012.07.015 22917990 PMC3577975

[20] LaDisa JF Jr Dholakia RJ Figueroa CA Vignon-Clementel IE Chan FP Samyn MM. Computational simulations demonstrate altered wall shear stress in aortic coarctation patients treated by resection with end-to-end anastomosis. Congenit Heart Dis. (2011) 6:432–43. 10.1111/j.1747-0803.2011.00553.x 21801315 PMC3208403

[21] Caimi A Pasquali M Sturla F Pluchinotta FR Giugno L Carminati M. Prediction of post-stenting biomechanics in coarcted aortas: a pilot finite element study. J Biomech. (2020) 105:109796. 10.1016/j.jbiomech.2020.109796 32423542

[22] Guillot M Ascuitto R Ross-Ascuitto N Mallula K Siwik E. Computational fluid dynamics simulations as a complementary study for transcatheter endovascular stent implantation for re-coarctation of the aorta associated with minimal pressure drop: an aneurysmal ductal ampulla with aortic isthmus narrowing. Cardiol Young. (2019) 29:768–76. 10.1017/S1047951119000751 31198121

[23] Tossas-Betancourt C van Bakel TM Arthurs CJ Coleman DM Eliason JL Figueroa CA. Computational analysis of renal artery flow characteristics by modeling aortoplasty and aortic bypass interventions for abdominal aortic coarctation. J Vasc Surg. (2020) 71:505–16. e4. 10.1016/j.jvs.2019.02.063 31153701 PMC8409007

[24] Yap C-H Dasi LP Yoganathan AP. Dynamic hemodynamic energy loss in normal and stenosed aortic valves. J Biomech Eng. (2010) 132:021005. 10.1115/1.4000874 20370242

[25] Whitehead KK Pekkan K Kitajima HD Paridon SM Yoganathan AP Fogel MA. Non-linear power loss during exercise in single-ventricle patients after the fontan: insights from computational fluid dynamics. Circulation. (2007) 116:I165–71. 10.1161/CIRCULATIONAHA.106.680827 17846299

[26] Sophocleous F Bone A Shearn AIU Velasco Forte MN Bruse JL Caputo M. Feasibility of a longitudinal statistical atlas model to study aortic growth in congenital heart disease. Comput Biol Med. (2022) 144:105326. 10.1016/j.compbiomed.2022.105326 35245697

[27] Olivieri LJ de Zélicourt DA Haggerty CM Ratnayaka K Cross RR Yoganathan AP. Hemodynamic modeling of surgically repaired coarctation of the aorta. Cardiovasc Eng Technol. (2011) 2:288–95. 10.1007/s13239-011-0059-1 22347895 PMC3279918

[28] LaDisa JF Alberto Figueroa C Vignon-Clementel IE Jin Kim H Xiao N Ellwein LM. Computational simulations for aortic coarctation: representative results from a sampling of patients. J Biomech Eng. (2011) 133:091008. 10.1115/1.4004996 22010743 PMC3705983

[29] Ntsinjana HN Biglino G Capelli C Tann O Giardini A Derrick G. Aortic arch shape is not associated with hypertensive response to exercise in patients with repaired congenital heart diseases. J Cardiovasc Magn Res. (2013) 15:1–7. 10.1186/1532-429X-15-101 24219806 PMC3833644

[30] Egbe AC Allison TG Ammash NM. Mild coarctation of aorta is an independent risk factor for exercise-induced hypertension. Hypertension. (2019) 74:1484–9. 10.1161/HYPERTENSIONAHA.119.13726 31630577

[31] Curtis SL Bradley M Wilde P Aw J Chakrabarti S Hamilton M. Results of screening for intracranial aneurysms in patients with coarctation of the aorta. Am J Neuroradiol. (2012) 33:1182–86. 10.3174/ajnr.A2915 22322607 PMC8013223

